# Editorial: Neuropharmacological intervention for severe mental illness and suicide prevention

**DOI:** 10.3389/fphar.2025.1599083

**Published:** 2025-04-08

**Authors:** Ewa Litwa, Stefano Comai, Agnieszka Zelek-Molik

**Affiliations:** ^1^ Department of Pharmacology, Maj Institute of Pharmacology, Polish Academy of Sciences, Krakow, Poland; ^2^ Department of Pharmaceutical and Pharmacological Sciences, University of Padua, Padua, Italy; ^3^ Department of Psychiatry, McGill University, Montreal, QC, Canada; ^4^ Department of Brain Biochemistry, Maj Institute of Pharmacology, Polish Academy of Sciences, Krakow, Poland

**Keywords:** arteminisin, atorvastatin, dezocine, ferroptosis, neuroinflammation, neurotrophic factors, treatment-resistant depression

Severe mental illnesses (SMI), including major depressive disorder, bipolar disorder, and schizophrenia, are leading contributors to disability worldwide and are associated with an elevated risk of suicide. Despite advancements in pharmacological and non-pharmacological interventions, a substantial proportion of individuals with SMI continue to experience persistent symptoms, treatment resistance, and significant comorbidities. These challenges underscore the critical need for novel and more effective therapeutic strategies. Understanding how different medications affect both core psychiatric symptoms and suicide risk remains a key area of research.

The collected publications present data derived from human subjects with mental illnesses, including post-mortem analyses, as well as from preclinical animal models. These translational studies investigate potential pathophysiological mechanisms and novel pharmacological interventions for SMI, effectively bridging the gap between preclinical findings and clinical applications. Additionally, the Research Topic also includes comprehensive reviews that synthesize current knowledge and outline future directions for innovative pharmacological strategies in treating SMI.

Among preclinical findings, Liu et al. employed a single prolonged stress model in rats to investigate the effects of artemisinin, a natural compound derived from *Artemisia annua*, on PTSD-related cognitive and social deficits. The study demonstrated that systemic administration of artemisinin alleviated behavioral impairments, with ultrastructural and molecular imaging data suggesting that its therapeutic effects are mediated by the restoration of synaptic plasticity and the inhibition of neuronal apoptosis in the hippocampus. Zelek-Molik et al. utilized a restraint stress (RS) model in rats to examine the impact of betaxolol, a selective β1-adrenergic receptor antagonist, on stress-induced alterations in glutamatergic signaling. While chronic RS was associated with GluN2B downregulation in the frontal cortex, pharmacological experiments did not support a direct role of β1-adrenergic receptor modulation in this process, implicating alternative pathways in stress-induced neurobiological changes. Further, Espinosa et al. explored the role of purinergic receptor modulation in temporal lobe epilepsy using *in vivo* electrophysiology in a mouse model. Although ATP receptors, particularly P2XRs, have been implicated in epileptic processes, their precise role in seizure pathophysiology remains unclear. The study documented that systemic administration of TNP-ATP reduced the amplitude and frequency of interictal discharges in the hippocampus. Notably, blocking P2XRs also improved cognitive performance and enhanced phase coherence for both slow (theta) and fast (gamma) oscillations recorded in the hippocampal CA1 region and prefrontal cortex, suggesting potential cognitive benefits of purinergic modulation in epilepsy-related neuropsychiatric symptoms.

Among human studies, Aldossary et al. investigated the adjunctive use of high-dose atorvastatin with fluoxetine in major depressive disorder, revealing modulation of the AMPK/NLRP3 and IL-6/STAT3 pathways, key regulators of neuroinflammation increasingly associated with mood regulation and suicidality. Qi et al. examined the pharmacokinetic consequences of smoking cessation in patients treated with clozapine, reinforcing the necessity of therapeutic drug monitoring to optimize treatment efficacy and minimize adverse effects in this vulnerable population. Wang et al. reported rapid and sustained antidepressant effects of dezocine, an opioid analgesic, in a patient with treatment-resistant depression, highlighting opioid receptor modulation as a potential therapeutic avenue for mood disorders and suicide prevention. Additionally, post-mortem studies by De Simone et al. analyzed the expression of brain-derived neurotrophic factor (BDNF) and glial cell line-derived neurotrophic factor (GDNF) in various brain regions of suicide victims with a clinical history of depression. Their findings indicated decreased BDNF and increased GDNF levels in multiple brain regions of suicide victims compared to individuals who died from natural causes, suggesting that these neurotrophic factors could serve as biomarkers for suicide risk and potential targets for specific pharmacological treatments of depression.

In a review article, Liu et al. explored pharmacological agents that mimic the antidepressant effects of exercise, emphasizing the role of irisin, a newly discovered exercise-induced myokine, and its precursor protein, fibronectin type III domain-containing protein 5 (FNDC5). Given the well-documented benefits of physical activity in alleviating depressive symptoms without the adverse effects associated with pharmacotherapy, the review underscores the potential of targeting exercise-related molecular pathways in depression treatment. Zhao et al. examined ferroptosis and its relevance to cardio-cerebrovascular diseases associated with depression. The authors highlight the multi-targeted therapeutic potential of traditional Chinese medicine and proposed GPX4 and Nrf2 as converging molecular targets implicated in both psychiatric and inflammatory diseases. Zelek-Molik and Litwa reviewed recent advances in antidepressant mechanism research, focusing on treatment-resistant depression, which is characterized by poorer outcomes, increased suicide risk, and heightened psychiatric comorbidities. The authors emphasized the necessity of multidimensional therapeutic strategies that account for the heterogeneity of mood disorders and the complex interplay of neurobiological systems. Despite significant advances, the pathophysiology of SMI remains idiopathic and incompletely understood, complicating the development of universally effective treatments.

As illustrated by the contributions in this Research Topic, mood disorders comorbidities are highly complex, and extend beyond the nervous system, involving intricate interactions among neural, immune, endocrine, metabolic, and microbiome-related processes. Ongoing research into novel pharmacological agents, multimodal treatment strategies, and precision medicine approaches represents the most promising pathway toward advancing the treatment of depression and prevent risky behaviors. These findings underscore the importance of personalized interventions tailored to address both core psychiatric symptoms and associated risk factors. Future studies should further integrate neurobiological, genetic, and environmental perspectives to refine pharmacological treatment paradigms and enhance patient outcomes in SMI and comorbidities.

